# Ingestion and Pharyngeal Trauma Causing Secondary Retropharyngeal Abscess in Five Adult Patients

**DOI:** 10.1155/2012/943090

**Published:** 2012-11-25

**Authors:** Sudhir B. Sharma, Paul Hong

**Affiliations:** ^1^Department of Surgery, Georgetown Public Hospital Corporation, University of Guyana, Georgetown, Guyana; ^2^Division of Otolaryngology-Head and Neck Surgery, Department of Surgery, IWK Health Centre, Dalhousie University, P.O. Box 9700, Halifax, NS, Canada B3K 6R8; ^3^School of Human Communication Disorders, Dalhousie University, Halifax, NS, Canada B3H 4R2

## Abstract

Retropharyngeal abscess most commonly occurs in children. When present in adults the clinical features may not be typical, and associated immunosuppression or local trauma can be part of the presentation. We present a case series of five adult patients who developed foreign body ingestion trauma associated retropharyngeal abscess. The unusual pearls of each case, along with their outcomes, are discussed. Pertinent information for the emergency medicine physician regarding retropharyngeal abscess is presented as well.

## 1. Introduction

Retropharyngeal abscess (RPA) usually occurs in children and is uncommonly reported in adults [1–3]. Clinically, RPA may pose a diagnostic challenge because of its infrequent occurrence and its variable presentations. Most common features include sore throats, neck stiffness, fever, dysphagia/odynophagia, and rarely respiratory distress and stridor [1]. On examination, a retropharyngeal or parapharyngeal prominence may be observed but adults may have trismus and children may not be cooperative, thereby preventing an adequate intraoral visualization. Other associated or preceding clinical entities may include pharyngotonsillitis, peritonsillar cellulitis/abscess, parapharyngeal cellulitis/abscess, and rarely complicated otitis media [1, 4].

Due to the possible airway compromise and spreading infections (mediastinitis, aspiration pneumonia, epidural abscess, jugular venous thrombosis, necrotizing fasciitis, sepsis, and carotid artery erosion), mortality can occur but is rare in the developing world [3, 5]. Morbidity with cranial neuropathy (CN VII, IX-XII), sympathetic plexus injury, osteomyelitis, and septic emboli formation from internal jugular vein thrombophlebitis (Lemierre syndrome) can complicate untreated or aggressive RPA as well [5, 6].

Initial management involves symptomatic relief, especially in regards to oxygen supplementation and securing of the airway in severe cases. However, most presentations will not be critical enough for such emergent measures to be instituted; rather supportive management with antipyretics, analgesics, and intravenous antibiotics will suffice in some patients with RPA [1, 2, 5]. For the refractory cases and for those who present with large abscesses, the standard management involves incision and drainage [2].

As mentioned above, RPA tends to occur mostly in children [1–3] and due to the rarity of this condition in adults, the definitive diagnosis may be delayed in some adult patients [7, 8]. Furthermore, some adults may not always present with the typical features of RPA that is observed in children. 

 In this paper, we describe a case series of five adults with RPA secondary to foreign body induced pharyngeal trauma. Details of the patient characteristics, clinical presentations, and management are described.

## 2. Case Series

**Case 1**

**Figure 1:**
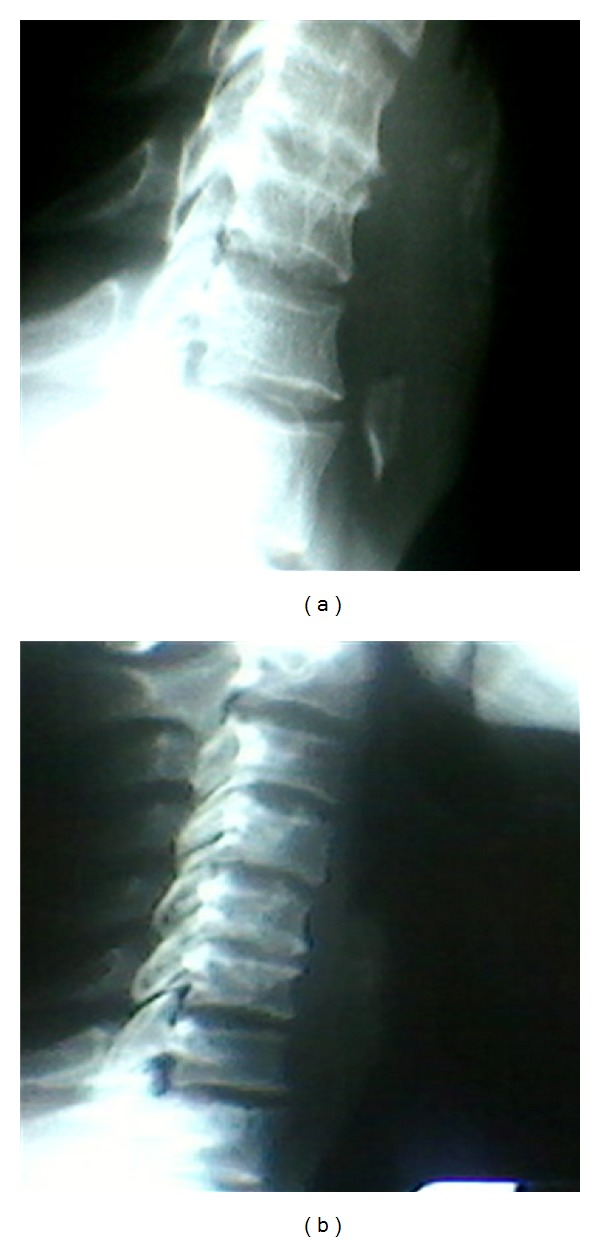
Lateral neck radiographic image of a chicken bone embedded in the retropharyngeal space (a) and after endoscopic removal of foreign body (b).

A 43-year-old male presented to the emergency department with odynophagia for 7 days, following ingestion of a chicken bone. The examination revealed restricted range of neck motion but no intraoral mass or other abnormalities were noted. The patient was afebrile as well. A lateral radiographic image of the neck demonstrated a foreign body in the retropharyngeal region at the level of 7th cervical vertebral bone (Figure 1).

Surgical consultation led to endoscopic removal of the foreign body, which turned out to be a chicken bone, surrounded by granulation tissue and approximately 2 to 4 mL of purulent discharge was elicited. A repeat radiograph done 48 hours after the procedure showed the absence of foreign body (Figure 1). The patient was discharged home on oral antibiotic therapy and did well with no complications.

**Case 2**

**Figure 2:**
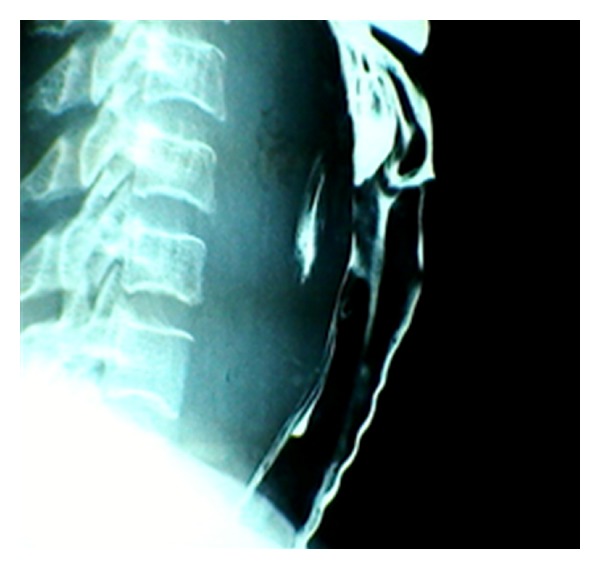
Lateral neck radiographic image (barium swallow study) of a patient who developed retropharyngeal abscess secondary to fish bone ingestion trauma.

A 45-year-old female with diabetes mellitus, developed odynophagia after suspected fish bone ingestion, one month prior to presentation. She had a history of reduced oral intake and several days of fever. On examination, she was dehydrated and an intraoral prominence was noted in the oropharynx. The lateral radiograph showed retropharyngeal thickening (Figure 2) and surgical consultation was made. Initially, intraoral drainage was performed, which produced about 10 mL of purulent discharge. She was admitted and placed on intravenous antibiotics, in addition to tight glucose control regimen. The culture was negative with no bacterial growth.

The symptoms persisted in the hospital and subsequently a transcervical drainage was performed with placement of an external drain. The drain was removed 72 hours later when the abscess and symptoms resolved. The patient was discharged home with oral antibiotics and did well.

**Case 3**

**Figure 3:**
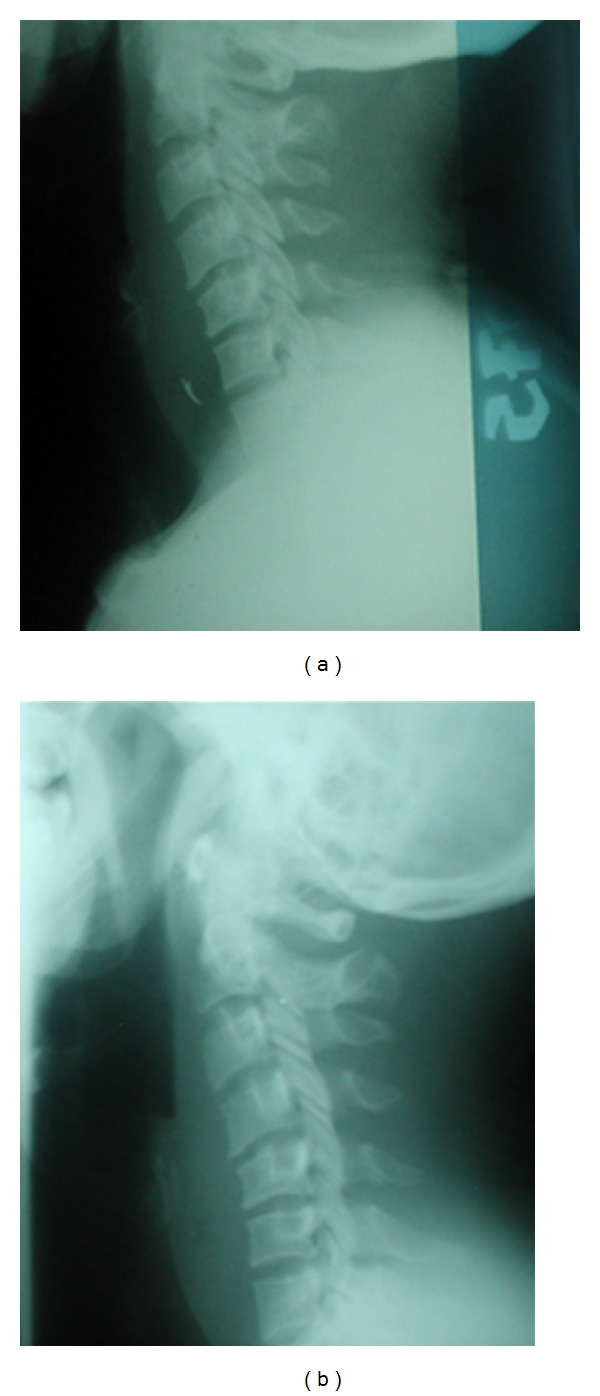
Lateral neck radiographic image of a foreign body in the retropharynx (a) and immediately after endoscopic removal (b).

**Figure 4:**
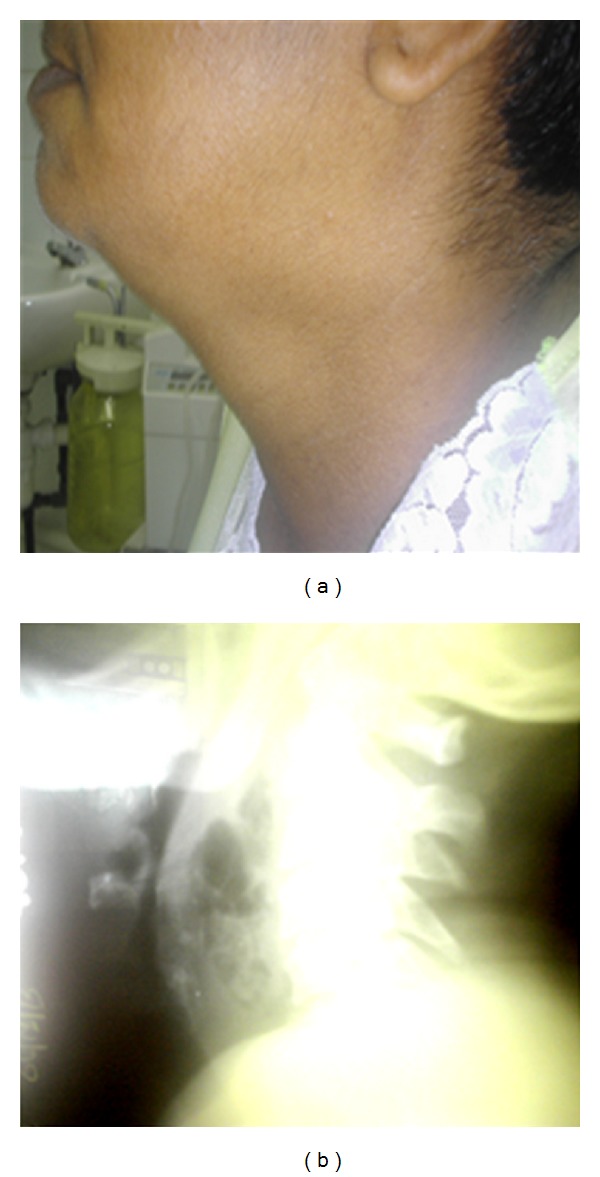
A lateral photograph demonstrating the central neck fullness secondary to retropharyngeal and neck inflammation (a). The same patient's radiographic image showing a significant retropharyngeal abscess (b).

A 46-year-old female presented to the emergency department with the onset of painful swallowing, after ingesting a fish bone, a day earlier. No other local or systemic symptoms were present. The lateral radiograph of the neck showed a foreign body in the retropharyngeal space (Figure 3), which was removed by the consulting otolaryngologist. She was discharged home on oral antibiotics but returned to the hospital after a week of worsening odynophagia, fever, and central painful neck fullness (Figure 4). A repeat radiograph showed a large RPA, which required urgent incision and drainage. The culture results showed Gram-positive alpha hemolytic streptococci and the patient was discharged home on oral antibiotics after 2-days of in-hospital observation with intravenous antibiotic therapy.

**Case 4**

**Figure 5:**
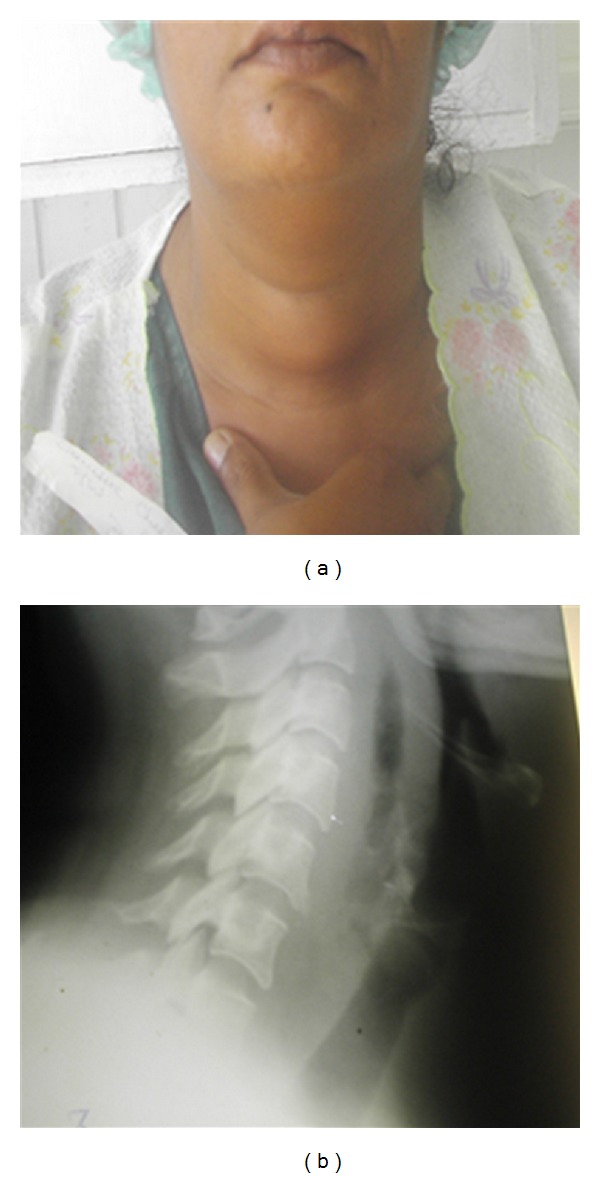
A photograph demonstrating the central neck swelling (a). Lateral radiograph of the same patient showing extensive retropharyngeal thickening with anterior hyperdensity.

A 39-year-old female, with diabetes mellitus, presented with a 3-day history of fever, neck swelling, and odynophagia. She reported self-induced trauma to the pharynx by her fingers, trying to feel for a suspected chicken bone. A swelling, with overlying mild erythema, was noted in the central portion of her neck, around the area of the thyroid cartilage (Figure 5). No intraoral mass was noted. Radiographic image of the neck demonstrated retropharyngeal thickening with anterior extension (Figure 5).

The RPA was drained through a transcervical approach and the culture analysis showed no growth. She was discharged home on postoperative day 3 with oral antibiotics.

**Case 5**

**Figure 6:**
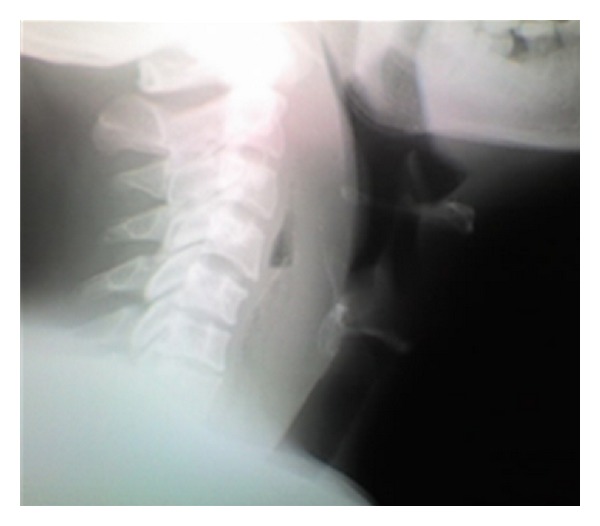
Lateral neck radiographic image of a patient who developed retropharyngeal abscess secondary to fish bone ingestion trauma.

A 43-year-old man with sore throat and painful swallowing presented to the emergency department. He reported swallowing a fish bone 6 days prior to presentation. On examination, he had a painful right neck swelling, lateral, and inferior to the thyroid cartilage. Radiograph showed a foreign body embedded at the upper posterior cervical esophagus with significant retropharyngeal thickening (Figure 6).

**Figure 7:**
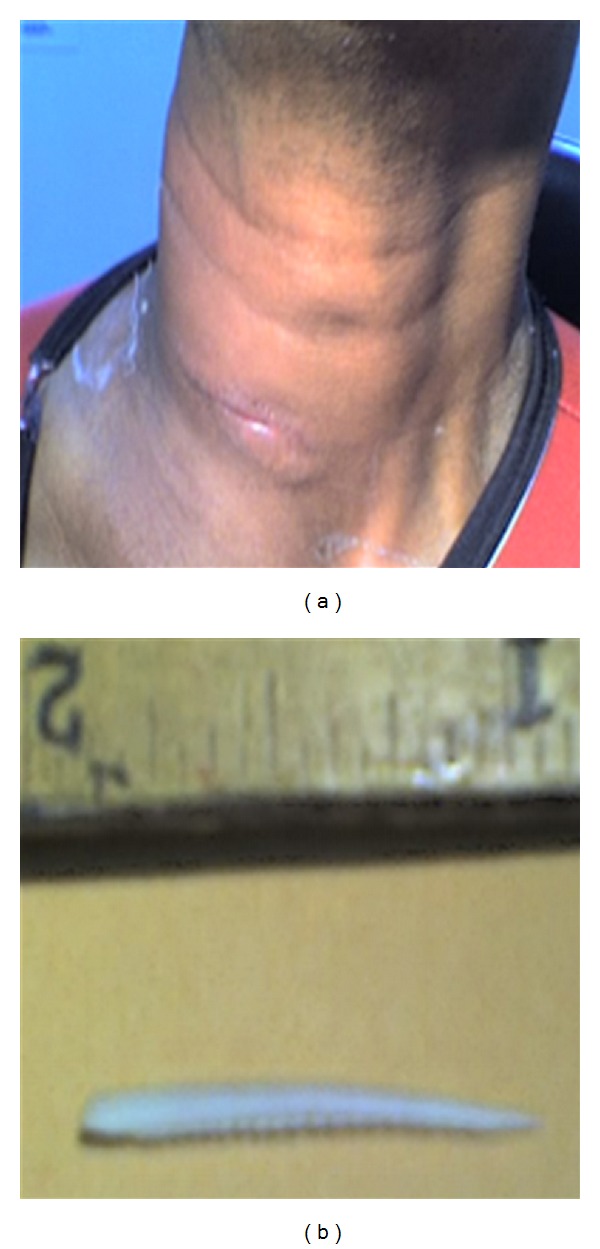
A photograph of the neck wound of a patient who underwent transcervical drainage procedure for a retropharyngeal abscess (a). A small fish bone was removed through the wound (b).

A transcervical drainage was performed by the consulting surgeon but no foreign body was identified. No microorganisms were grown on culture analysis. On the second postoperative day, the foreign body (fish bone), along with some discharge, was elicited through the partially open neck wound (Figure 7). The patient was discharged home 4 days after the drainage procedure with oral antibiotics. No further complications were reported.

## 3. Discussion

The five cases described above presented to the Georgetown Public Hospital Corporation in Georgetown, Guyana during the time period between 2004 and 2011. This suggests that RPA is not only a disease of childhood but should be considered in adults presenting with upper aerodigestive tract associated symptoms [9].

The differential diagnosis includes epiglottitis, supraglottitis, angioedema, croup, caustic ingestion, foreign body ingestion, and mediastinitis [1, 2]. Definitive diagnosis usually requires imaging, such as lateral neck radiography and computed tomography (CT). On CT images, the RPA usually appears as a hypodense lesion with peripheral rim enhancement, in addition to soft-tissue thickening, obliterated fat planes and mass effect to surrounding tissues [2, 3]. The advantage of CT is that an abscess can be differentiated from cellulitis or early abscess (phlegmon). This information is valuable since an operation (incision and drainage) may not be needed for cases of early abscess or cellulitis. 

As mentioned above, management starts by ensuring the presence of an adequate airway. For severe cases with upper airway compromise, the emergency department physician may be required to perform endotracheal intubation. Yet, one must be aware that intubation can be a challenge due to the swollen pharyngeal walls, cervical spine rigidity, and trismus. Furthermore, traumatic rupture of the RPA has been reported with intubation and rarely, this had led to the development of mediastinitis [1, 3]. Therefore, a better option may be to secure the airway via needle cricothyrotomy or tracheostomy. Other initial supportive therapy includes hydration and management of concurrent medical diseases (e.g., diabetes) [10].

For select cases of small abscesses and retropharyngeal cellulitis, intravenous antibiotics may be instituted as the sole therapy, and complete resolution may occur [1, 3]. Broad-spectrum antibiotics, with optimal oral anaerobic coverage, should be selected as the initial medication (clindamycin, ampicillin/sulbactam, cefuroxime) [2, 3]. However, these patients should be observed closely since for those who do not show improvements over 48 hours of antibiotic therapy, incision and drainage procedure should be performed. 

Incision and drainage procedure mainly involves two approaches: (1) intraoral and (2) transcervical. There are advantages and disadvantages of both techniques and many studies have reported success with both methods [1–3, 9]. Some authors have suggested attempting the less-invasive intraoral approach first, followed by the transcervical incision and drainage for recurrent/residual diseases [3, 11]. A similar approach was taken in one of our patients (Case  2), who underwent an intraoral incision and drainage, followed by a transcervical approach. 

Significant complications can occur secondary to RPA (see above) but the overall prognosis is generally favorable if identified early and managed appropriately. In a large series of RPA in the United States, no fatality was reported [12], while in other studies, the mortality rate was 1% to 2.6% [13, 14]. Primary cause of mortality was sepsis with multiorgan failure. The morbidity rate has been reduced dramatically due to the advent of advanced microbiology and broad-spectrum antibiotics, development of sophisticated diagnostic tools, improved surgical skills, and improved medical care and awareness of physicians.

## 4. Conclusion

Five cases of adult RPA secondary to ingestion trauma have been described in the current paper. Typically, a food substance with sharp and rigid edges can get embedded into the retropharyngeal soft-tissue and cause local cellulitis and abscess formation.

Although RPA is mostly a childhood disease, adults can also be affected. Therefore, it is important for the emergency medicine physician to have a high degree of suspicion when adults present with upper aerodigestive tract associated complaints.
